# Ischemia-Reperfusion Lung Injury Is Attenuated in MyD88-Deficient Mice

**DOI:** 10.1371/journal.pone.0077123

**Published:** 2013-10-11

**Authors:** William A. Altemeier, W. Conrad Liles, Ana Villagra-Garcia, Gustavo Matute-Bello, Robb W. Glenny

**Affiliations:** 1 Center for Lung Biology, University of Washington, Seattle, WA, United States of America; 2 Department of Medicine, University of Washington, Seattle, WA, United States of America; 3 Department of Medicine, McLaughlin-Rotman Centre for Global Health, Toronto General Research Institute, University of Toronto, Toronto, Ontario, Canada; 4 Department of Medicine, McLaughlin-Rotman Centre for Global Health, Toronto General Research Institute, University of Toronto, Toronto, Ontario, Canada; 5 Department of Physiology and Biophysics, University of Washington, Seattle, WA, United States of America; University of Colorado Denver, United States of America

## Abstract

Ischemia-reperfusion lung injury is a common cause of acute morbidity and mortality in lung transplant recipients and has been associated with subsequent development of bronchiolitis obliterans syndrome. Recognition of endogenous ligands released during cellular injury (damage-associated molecular patterns; DAMPs) by Toll-like receptors (TLRs), especially TLR4, has increasingly been recognized as a mechanism for inflammation resulting from tissue damage. TLR4 is implicated in the pathogenesis of ischemia-reperfusion injury of multiple organs including heart, liver, kidney and lung. Additionally, activation of TLRs other than TLR4 by DAMPs has been identified in tissues other than the lung. Because all known TLRs, with the exception of TLR3, signal via the MyD88 adapter protein, we hypothesized that lung ischemia-reperfusion injury was mediated by MyD88-dependent signaling. To test this hypothesis, we subjected C57BL/6 wildtype, *Myd88* ^*-/-*^, and *Tlr4* ^*-/-*^ mice to 1 hr of left lung warm ischemia followed by 4 hr of reperfusion. We found that *Myd88* ^*-/-*^ mice had significantly less MCP-1/CCL2 in the left lung following ischemia-reperfusion as compared with wildtype mice. This difference was associated with dramatically reduced lung permeability. Interestingly, *Tlr4* ^*-/-*^ mice had only partial protection from ischemia-reperfusion as compared to *Myd88* ^*-/-*^ mice, implicating other MyD88-dependent pathways in lung injury following ischemia-reperfusion. We also found that left lung ischemia-reperfusion caused remote inflammation in the right lung. Finally, using chimeric mice with MyD88 expression restricted to either myeloid or non-myeloid cells, we found that MyD88-dependent signaling in myeloid cells was necessary for ischemia-reperfusion induced lung permeability. We conclude that MyD88-dependent signaling through multiple receptors is important in the pathogenesis of acute lung inflammation and injury following ischemia and reperfusion.

## Introduction

Lung transplantation is employed with increasing frequency to treat a variety of end-stage lung diseases including emphysema, pulmonary fibrosis, and cystic fibrosis. However, despite recent advances in organ preservation technique, primary graft dysfunction from ischemia-reperfusion injury remains the major cause of morbidity and mortality in the first 72 hours following lung transplantation [1,2]. Additionally, ischemia-reperfusion lung injury is associated with an increased risk of bronchiolitis obliterans syndrome, the primary cause of late death following lung transplantation [3]. Therefore, understanding the mechanisms by which ischemia-reperfusion lung injury develops is critical to extending survival following lung transplantation.

Although many mechanisms contribute to the pathogenesis of ischemia-reperfusion injury, activation of pro-inflammatory pathways plays a vital role. Studies in a rat model of left lung ischemia-reperfusion found attenuated alveolar-capillary barrier dysfunction after four hours of reperfusion in animals with antibody-mediated neutrophil depletion [4]. Consistent with an important role for neutrophils in ischemia-reperfusion lung injury, CXC chemokines that promote neutrophil chemotaxis and activation are elevated in bronchoalveolar lavage fluid [5] and in lung tissue [6] of patients with primary graft dysfunction, following lung transplantation. In a rat model of lung transplantation, blocking the CXC chemokine receptor, CXCR2, with a monoclonal antibody results in dramatic attenuation of both neutrophil recruitment and lung injury [5]. In contrast, the CXC chemokine, IL-8, is not elevated in the serum of patients with primary graft dysfunction; however, both the monocyte chemokine, MCP-1/CCL2, and the lymphocyte chemokine, IP-10/CXCL10, are increased [7]. A second study confirmed elevated serum MCP-1/CCL2 as an independent predictor of primary graft dysfunction, following lung transplantation [8]. Most chemokines and other early response pro-inflammatory cytokines are primarily regulated at the transcriptional level, with the transcription factors, NF-κB and AP-1, playing critical roles [9]. Both NF-κB and AP-1 are activated in ischemia-reperfusion lung injury, and pharmacologic disruption of both of these pathways attenuates the severity of injury [10,11]. 

The mechanisms by which these pro-inflammatory pathways are activated are incompletely understood. NF-κB and AP-1 are both redox sensitive transcription factors [12,13], and oxidative stress is thought to be an important mechanism by which inflammation and injury develop after lung ischemia-reperfusion [1,14,15]. However, an alternative mechanism for activation of pro-inflammatory pathways is via MyD88-dependent signaling pathways. MyD88 (myeloid differentiation response gene 88) is a widely expressed adapter molecule through which all members of the Toll-like receptor (TLR)/IL-1 receptor family, with the exception of TLR3, initiate intracellular kinase activation resulting, ultimately, in NF-κB and AP-1-mediated gene transcription [16,17]. Although a variety of pathogen-associated molecular patterns (PAMPs) are the primary ligands for Toll-like receptors (TLRs), endogenous ligands released during cellular injury (damage-associated molecular patterns, DAMPs), have been increasingly recognized as potential ligands for multiple different TLRs [18]. TLR4 has been the most widely studied receptor for DAMPs and contributes to ischemia-reperfusion injury in kidney [19], liver [20,21], and heart [22-24]. In the lung, Shimamoto et al. reported that *Tlr4* ^*-/-*^ mice have partially reduced cytokine release, leukocyte recruitment, and permeability associated with attenuated NF-κB and AP-1 binding activity following one hour of left lung ischemia and three hours of reperfusion [25]. A subsequent study reported that HeJ mice, which lack functional TLR4, have reduced permeability following left lung ischemia and reperfusion [26]. 

The role of TLRs other than TLR4 in lung ischemia-reperfusion injury has not been described; however, multiple TLRs have been reported to modulate injury following ischemia-reperfusion in other organs. TLR2 signaling contributes to renal inflammation and injury following ischemia-reperfusion [27], and inhibition of TLR2 improves sub-acute graft function in a murine model of kidney transplantation [28]. Infusion of a blocking TLR2 antibody reduces cytokine production, leukocyte influx, and infarct size in a murine model of myocardial ischemia-reperfusion [29]. In contrast, treatment with TLR2 agonists was recently reported to reduce infarct size following cerebral ischemia-reperfusion [30]. TLR9 activation has been reported to both contribute to liver ischemia-reperfusion injury and to preserve left ventricular ejection fraction following cardiac ischemia-reperfusion [31-33]. Together, these data suggest that multiple different TLRs may be activated by endogenous ligands or DAMPS released by cellular injury, following ischemia-reperfusion. Furthermore, the specific modulatory effect of individual TLR activation may vary in different tissue types. We therefore hypothesized that lung ischemia-reperfusion injury is mediated by multiple different MyD88-dependent receptors and that *Myd88* ^*-/-*^ mice will have less injury than either *Tlr4* ^*-/-*^ or normal mice, following lung ischemia and reperfusion. To test this hypothesis, we compared lung inflammation and injury in wildtype C57BL/6 mice, *Myd88* ^*-/-*^ mice, and *Tlr4* ^*-/-*^ mice following left lung warm ischemia and reperfusion. We found that *Myd88* ^*-/-*^ mice had reduced permeability and lower levels of the CC chemokine, MCP-1/CCL2, following lung ischemia-reperfusion. *Tlr4* ^*-/-*^ mice displayed an intermediate phenotype, suggesting that both TLR4-dependent and –independent pathways that utilize the MyD88 adapter protein contribute to acute lung inflammation and injury following ischemia and reperfusion.

## Methods

### Animals and Reagents

The University of Washington Animal Care and Use Committee approved all experiments. *Myd88* ^*-/-*^ and *Tlr4* ^*-/-*^ mice on a C57BL/6 background (>10 backcrosses) were generously provided by Dr. Thomas Hawn (University of Washington). Age-matched C57BL/6 mice were purchased from Jackson Laboratories (Hollister, CA). Mice were housed under specific-pathogen-free conditions for a minimum of 7 days prior to use.

Multiplex bead array reagents for detection of murine KC/CXCL1, MCP-1/CCL2, IL-6, and IL-1β and reagents for ELISA detection of human IL-8 were purchased from R & D Systems (Minneapolis, MN). Fluorescein-labeled 70kD dextran was purchased from Invitrogen Life Sciences (Carlsbad, CA). HEK293T cells that stably express murine TLR4, MD2, and CD14 and polymixin B were purchased from Invivogen (cat#293-mtlr4md2cd14; San Diego, CA). Lipopolysaccharide (LPS – *E. coli* serotype 011:B4) was purchased from List Biological Laboratories (Campbell, CA). All other chemicals were from Sigma-Aldrich (St. Louis, MO) unless otherwise specified.

### Lung Ischemia-Reperfusion Protocol

All instruments were sterilized prior to use and baked at 80°C overnight to decrease LPS contamination. The night before an experiment, each mouse was weighed and subcutaneously injected with 1 ml of sterile lactate Ringer’s solution, containing 5% dextrose (D5LR). The next morning, the mouse was anesthetized with 4% isoflurane for 5 min in an anesthesia chamber and then with 2% isoflurane via nose cone prior to intubation by tracheostomy with a blunt 20-gauge needle. The intubated mouse was connected to a ventilator (MiniVent, Harvard Apparatus, Holliston, MA) and mechanically ventilated with a tidal volume of 10 ml/kg, a respiratory rate of 150 breaths per minute, an end-expiratory pressure of 3-cmH_2_O, an inspired oxygen fraction of 0.4, and 1.5% isoflurane. A repeat subcutaneous injection of 1 ml D5LR was administered, and pancuronium (20 µl/gram body weight of a 0.1 µg/µl in D5LR) was given i.p. Pancuronium 10 µl/gram body weight was re-administered i.p. every 60 min for the duration of the experiment. Airway pressure and respiratory rate were continuously acquired (PowerLab, AD Instruments, Colorado Springs, CO). Mixed expired CO_2_ (CO_2_SMO Model 7100, Novametrix, Wallingford, CT) was continuously monitored to confirm continued animal survival.

After collection of baseline data, isoflurane was increased to 2% and the mouse was placed in a right lateral decubitus posture for left thoracotomy after prepping with alcohol. The left lung hilum was isolated with minimal manipulation of the lung, and a microvascular clamp placed across all hilar structures (bronchus, artery, and vein). The thoracotomy site was covered with a moist surgical gauze, the animal rotated back to supine position with isoflurane reduced to 1.5%, tidal volume decreased to 6 ml/kg and the respiratory rate increased to maintain expired CO_2_ at a similar level.

After 60-min, the left hilar clamp was removed, the tidal volume increased to 10 ml/kg, and the respiratory rate decreased to 150 breaths/minute. The left lung was recruited by placing the lung on 15-cmH_2_O for 30-sec. Mechanical ventilation was continued for an additional four hours of reperfusion.

Sham surgery mice were prepared in an identical manner. However, after thoracotomy, the left hilum was not cross-clamped and mechanical ventilation was not adjusted. Mechanical ventilation with tidal volumes of 10 ml/kg, a respiratory rate of 150 bpm, and an end-expiratory pressure of 3 cmH_2_O was continued for five hours following thoracotomy.

At the end of the experimental period, all mice were deeply anesthetized with isoflurane and euthanized by cardiac puncture and exsanguination. The left atrium was cut, and the pulmonary vasculature flushed via the right ventricle with 1-ml of 4°C saline. Right and left lungs were dissected free and separately homogenized (Omni International, Marietta, GA) on ice in 1-ml of H_2_O containing a mix of protease inhibitors (*mini*-Complete tablets, Roche Applied Science, Indianapolis, IN) for determination of cytokine concentration and myeloperoxidase (MPO) activity as detailed below.

### Cytokine and MPO Determination

Lung homogenate was divided into two aliquots for determination of cytokine concentrations and MPO activity. For cytokines, homogenate was combined with cytokine lysis buffer (final concentration 0.5% Triton-X-100, 150 mM NaCl, 15 mM Tris, 1 mM CaCl, and 1mM MgCl, pH 7.40), incubated on ice for 30-min, and spun at 10,000 x *g* and 4°C for 20-min. Supernatant was stored at -80°C for batch cytokine determination. Concentrations of KC/CXCL1, MCP-1/CCL2, IL-6, and IL‑1β, were determined in duplicate by multiplex immunoassay (Luminex 100, Austin, TX).

MPO activity was measured as previously described [34]. Lung homogenate was combined with MPO buffer (final concentration 40 mM potassium phosphate, 0.5% hexadecyltrimethyl ammonium bromide, 5 mM EDTA, pH 6.0). Homogenate in buffer was lysed using four 15-sec pulses of a 130 ultrasonic cell disruptor (Daigger, Vernon Hills, IL) and spun at 10,000 x *g* and 4°C for 10-min. Supernatant was stored at -80°C for batch MPO determination. For each sample, 200 µl of supernatant was combined with H_2_O_2_ in the presence of 0-dianisidine dihydrochloride (0.167 mg/ml) for 15-min. The reaction was terminated by the addition of 50 µl of 10% sodium azide, and optical density was determined at 570 nm. 

### Lung Permeability Measurements

In a separate set of experiments to determine lung permeability, mice underwent the same ischemia-reperfusion or sham surgery protocol. Three hours prior to euthanasia, each mouse was injected with 100 µl of a 10 µM solution of fluorescein-labeled 70kD dextran (FITC-dextran) in sterile phosphate-buffered saline (PBS) via the retro-orbital venous plexus. Mice were euthanized under deep anesthesia by cervical dislocation. A median sternotomy was performed and the right hilum clamped. The left lung was lavaged with three 0.5 ml aliquots of PBS containing 0.6 mM EDTA at 37°C. Subsequently, the left hilum was clamped, the right hilum released, and the right lung was lavaged in an identical manner. Collected lavage fluid for each lung was pooled and spun at 1500 x *g* at 4°C for 10-min, and the fluorescent intensity of the supernatant determined in a fluorimeter (Perkin-Elmer LS-50B, Wellesley, MA) using an excitation wavelength of 494 nm and emission wavelength of 521 nm. Supernatant was recovered and stored at -80°C for later determination of protein concentration by Bradford assay (Pierce Biotechnology, Rockford, IL) and of IgM concentration by ELISA (Bethyl Laboratories, Montgomery TX).

### Determination of TLR4 Ligand Presence in BAL Fluid and Serum

In a separate set of experiments, C57BL/6 mice were subjected to ischemia-reperfusion as detailed above. After 5-hrs, mice were euthanized by cardiac puncture and exsanguination under 5% isoflurane anesthesia. Following sternotomy, the left and right lungs were individually lavaged with three 0.5 ml aliquots of PBS. BAL fluid from each lung was pooled and spun along with the serum at 10,000 x *g* and 4°C for 10-min. Supernatant from the BAL fluid and the serum were stored at -80°C for subsequent testing. 

HEK293T cells, expressing murine TLR4, MD2, and CD14, were grown to confluence in a 96-well format according to the manufacturer’s instructions. To test for the presence of a TLR4-ligand, medium was changed for new medium mixed 1:1 with the test sample (BAL fluid or serum), and cells were incubated for 18-hrs. After 18-hrs, medium was removed and stored at -80°C for later determination of human IL-8 concentration by ELISA. All conditions were tested in duplicate in the presence or absence of polymixin B (50 µg/ml), which binds to LPS and prevents recognition by TLR4. Positive control wells were treated with LPS at 10 ng/ml with or without polymixin B. Negative control wells were treated with medium only.

### Contribution of Myeloid- vs. Non-myeloid-derived Cells to MyD88-dependent Ischemia-reperfusion Injury

To evaluate which cell types contribute to MyD88-dependent ischemia-reperfusion lung injury, we generated chimeric mice expressing MyD88 restricted to either myeloid-derived cells or non-myeloid-derived cells. Enrofloxacin was added to water bottles (40 mg/8 oz) three to five days prior to total body irradiation. On day 0, wildtype and 

*Myd88* ^*-/-*^ mice were subcutaneously injected with 25 mg/kg body weight of enrofloxacin (22.7 mg/ml) and irradiated with 900 cGy delivered at 17.2 cGy/min. The following day, bone marrow cells were collected from either a wildtype or *Myd88* ^*-/-*^ donor mouse as previously described [35]. Bone marrow cells were re-suspended in sterile PBS and injected at a concentration of 5 x 10^6^ cells in 100 µl PBS into the retro-orbital venous sinus of previously irradiated mice, using a sterile 25 gauge needle. Transplanted mice received subcutaneous injections of enrofloxacin 4 mg/kg (0.227 mg/ml in PBS) daily through day 7. Enrofloxacin was also included in the cage water for two weeks following transplantation. After 60 days, by which time alveolar macrophages are 100% donor-derived [35], mice were subjected to 1-hr of left hilar cross-clamping followed by 4-hr of reperfusion. At the conclusion of reperfusion, mice were euthanized by isoflurane overdose and exsanguination by cardiac puncture. The left lung was lavaged with three 0.5 ml aliquots of warm PBS containing 0.6 mM EDTA. The returned fluid was pooled, spun at 1500 x *g* and 4°C for 10-min and the supernatant collected for subsequent determination of total protein and IgM concentrations as described above. Genomic DNA was isolated from the collected whole blood and genotype of the circulating leukocytes confirmed by PCR.

### Statistical Analysis

All data are presented as mean ± SEM. Data were compared by 2-way ANOVA with the main effects being genotype or condition (ischemia-reperfusion vs. sham surgery). An interaction term was included in the model to test for differing responses to the two conditions among the genotypes. Airway pressure data were analyzed by repeated-measures ANOVA. Post-hoc comparisons among all genotypes for the sham surgery condition and for the ischemia-reperfusion condition were made using a Bonferroni correction. A P ≤ 0.05 was used to determine statistical significance.

## Results

**Figure 1:**
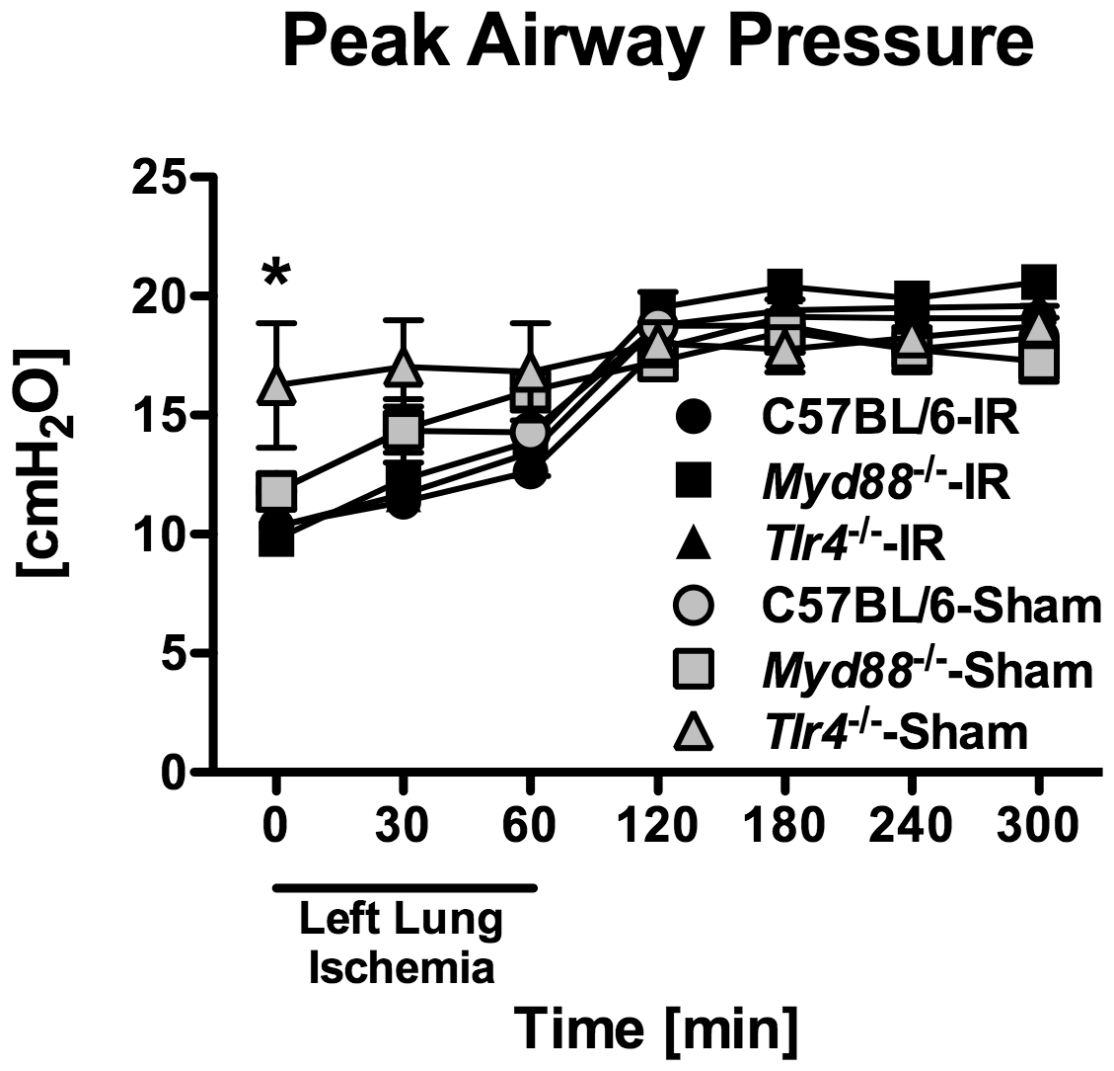
Peak airway pressures measured at various times during the experimental protocol for mice subjected to either 1 hr of left lung warm ischemia and 4 hr of reperfusion (-IR, n = 12/grp for C57BL/6 and *Tlr4* ^*-/-*^; n=10 for *Myd88* ^*-/-*^) or sham thoracotomy and 5 hr of mechanical ventilation (-Sham, n=4/grp). *p ≤ 0.05 between *MyD88* ^*-/-*^ and *Tlr4* ^*-/-*^ mice subjected to sham surgery at specific times.

A total of 18 wildtype C57BL/6 mice, 17 *Myd88* ^*-/-*^ mice, and 18 *Tlr4* ^*-/-*^ mice were studied following 1 hour of left lung ischemia and 4 hours of reperfusion. All mice survived the full reperfusion period. One *Myd88* ^*-/-*^ mouse and one *Tlr4* ^*-/-*^ mouse were excluded from further analysis because of an erroneous double injection of FITC-dextran to one mouse and no injection to the other mouse. Samples were collected from an additional four mice per genotype subjected to thoracotomy and five hours of mechanical ventilation without left lung ischemia. Airway pressures were similar among all groups over time (Figure 1). There were no statistical differences among any of the genotypes subjected to ischemia-reperfusion at any time. The only differences among the genotypes subjected to sham surgery were between *MyD88* ^*-/-*^ mice and *Tlr4* ^*-/-*^ mice at times 0-min and 30-min.

### Ischemia-reperfusion induced upregulation of MCP-1/CCL2 is attenuated in Myd88^-/-^ mice

**Figure 2:**
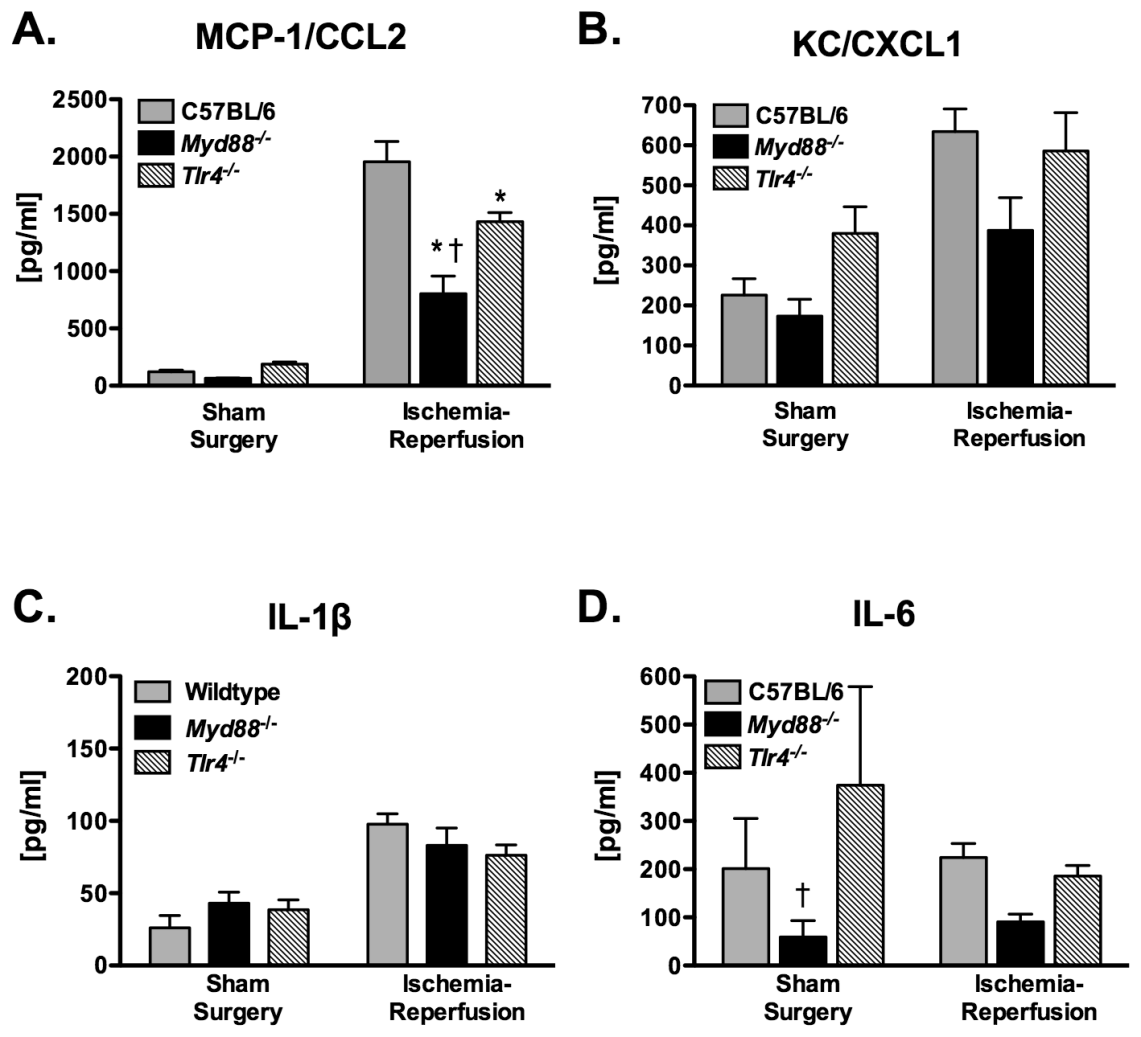
Cytokine concentrations from the left lung of mice subjected to 1 hr of left lung warm ischemia and 4 hr of reperfusion (n = 12/grp for C57BL/6 and *Tlr4* ^*-/-*^; n=10 for *Myd88* ^*-/-*^). Sham surgery mice (n = 4/grp) were subjected to the same procedure except for left lung ischemia. *p ≤ 0.05 when compared with C57BL/6 mice. ^†^p ≤ 0.05 when compared with *Tlr4* ^*-/-*^ mice.

In the first series of experiments, 12 wildtype, 12 *Tlr4* ^*-/-*^, and 10 *Myd88* ^*-/-*^ mice were subjected to one hour of left lung ischemia and 4 hours of reperfusion after which the lungs were recovered for cytokine and MPO determination. Ischemia-reperfusion resulted in a significant increase in MCP-1/CCL2 in left lung homogenate as compared to mice subjected to sham surgery, accounting for 48.5% of the total variance (Figure 2A, p < 0.001). Genotype also significantly influenced MCP-1/CCL2 expression and accounted for 7.5% of total variance (p = 0.005). The effect of genotype on response to ischemia-reperfusion (interaction effect) accounted for 6.0% of total variance (p = 0.01). Post-hoc comparison identified no differences among the genotypes in the sham surgery group. In contrast, *Myd88* ^*-/-*^ mice had the lowest concentration of MCP-1/CCL2 following ischemia-reperfusion and *Tlr4* ^*-/-*^ mice had an intermediate level.

Ischemia-reperfusion also increased expression of KC/CXCL1 (p = 0.002, Figure 2B) and IL1β (p < 0.001, Figure 2C); however, genotype did not have a statistically significant influence on expression of either cytokine. IL6 was not increased in the ischemia-reperfusion group as compared to the sham surgery group, although genotype had a statistically significant effect on IL6, accounting for 26.9% of overall variance (p = 0.002, Figure 2D) with the lowest levels observed in *Myd88* ^*-/-*^ mice.

**Figure 3:**
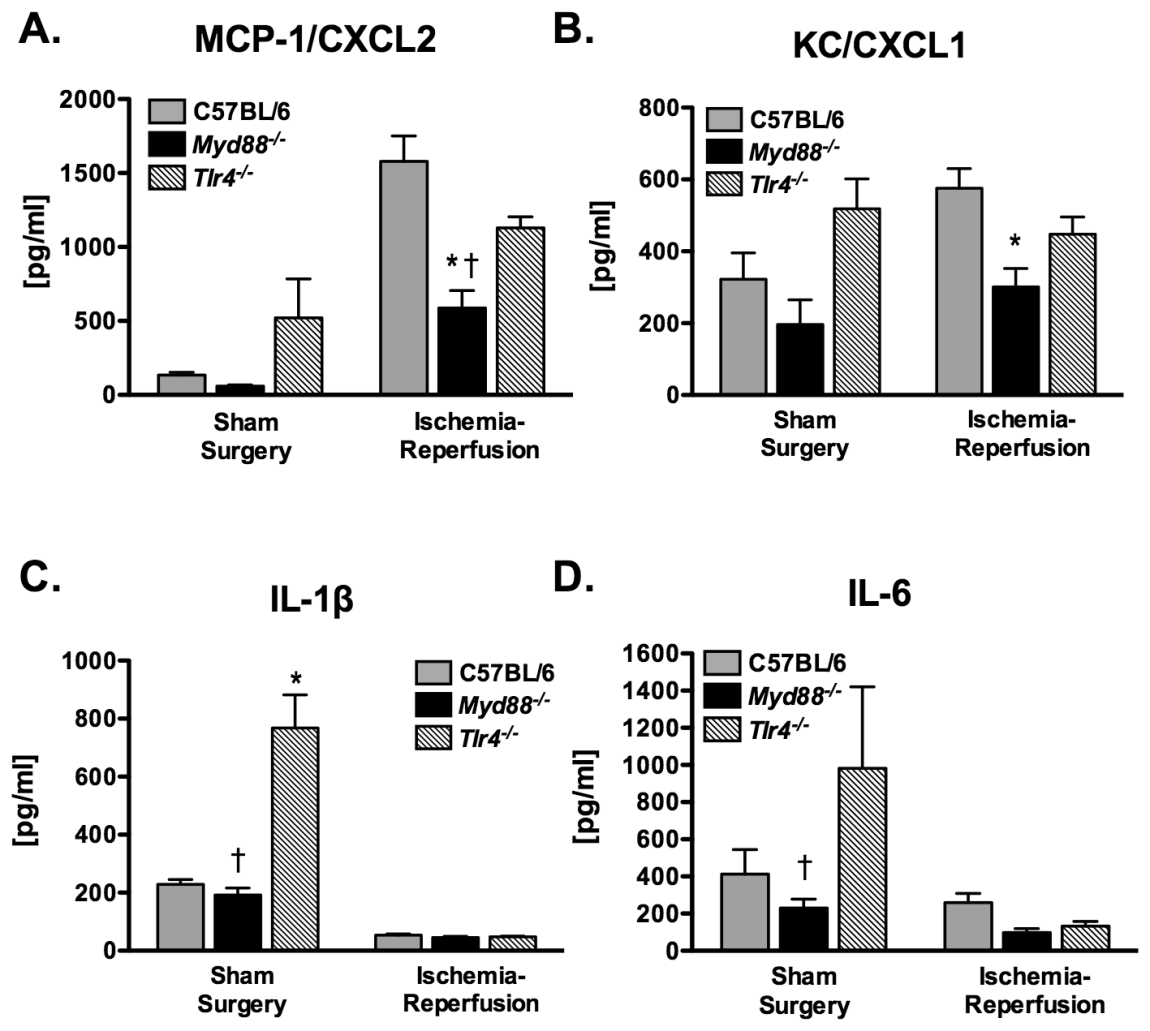
Cytokine concentrations from the right lung of mice subjected to 1 hr of left lung warm ischemia and 4 hr of reperfusion (n = 12/grp for C57BL/6 and *Tlr4* ^*-/-*^; n=10 for *Myd88* ^*-/-*^). Sham surgery mice (n = 4/grp) were not subjected to left lung ischemia. *p ≤ 0.05 when compared with the C57BL/6 mice. ^†^p ≤ 0.05 when compared with *Tlr4* ^*-/-*^ mice.

Interestingly, a similar pattern of MCP-1/CCL2 expression was also observed in the right lung. Left lung ischemia-reperfusion accounted for 33.9% of the total variance in right lung MCP-1/CCL2 levels (p < 0.001, Figure 3A). Genotype accounted for 10.5% of variance (p = 0.005) and the interaction between genotype and ischemia-reperfusion accounted for 7.7% of variance (p = 0.019). *Myd88* ^*-/-*^ mice had significantly less MCP-1/CCL2 expression as compared to both wildtype and *Tlr4* ^*-/-*^ mice following ischemia-reperfusion. Left lung ischemia-reperfusion did not have a significant effect on right lung KC/CXCL1 expression although genotype did (20.2% of total variance, p = 0.003, Figure 3B). Left lung ischemia-reperfusion was associated with reduced levels of both IL1β (p < 0.001, Figure 3C) and IL6 (p < 0.001, Figure 3D) in the right lung.

**Figure 4:**
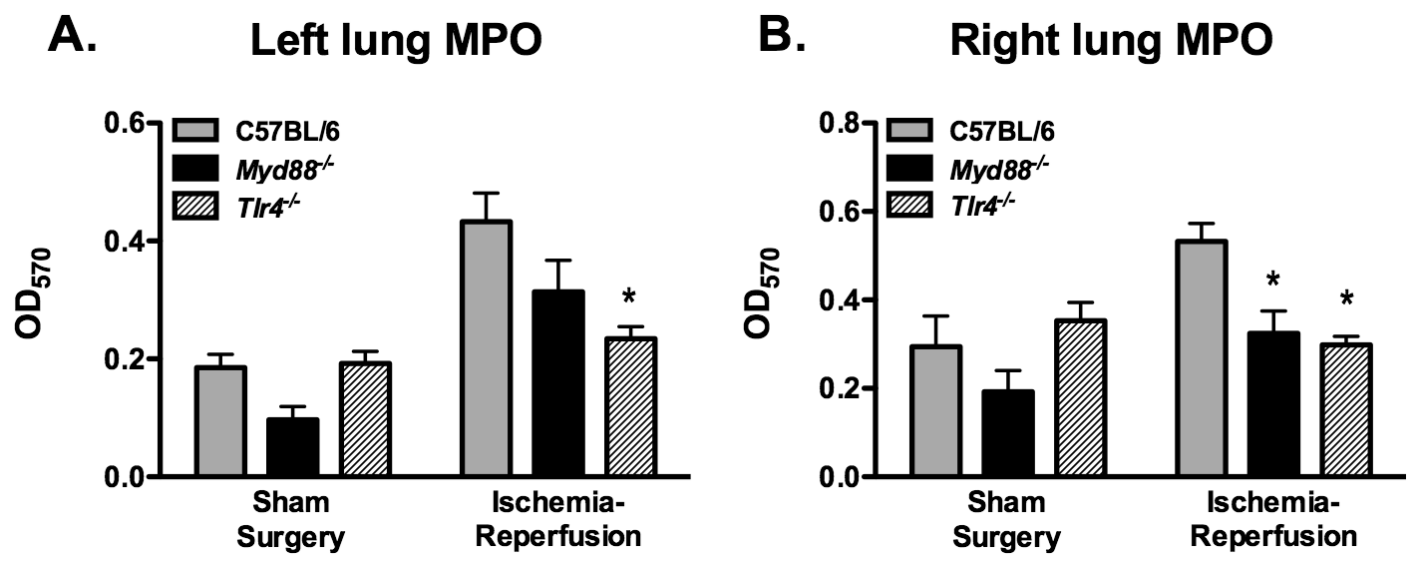
Myeloperoxidase activity in left lung (A) and right lung (B) homogenates of mice subjected to 1 hr of left lung warm ischemia and 4 hr of reperfusion (n = 12/grp for C57BL/6 and *Tlr4* ^*-/-*^; n=11 for *Myd88* ^*-/-*^). Sham surgery mice (n = 4/grp) were not subjected to left lung ischemia. *p ≤ 0.05 when compared with C57BL/6 mice.

Ischemia-reperfusion increased left lung MPO activity, accounting for 22.7% of total variance (p < 0.001, Figure 4A). No significant overall effect of genotype on MPO activity was observed; however, there was a trend toward a significant interaction effect between genotype and ischemia-reperfusion (p = 0.09). Post hoc comparisons revealed a statistically significant difference only between C57BL/6 mice and *Tlr4* ^*-/-*^ mice following ischemia-reperfusion. Right lung MPO activity was also influenced by left lung ischemia-reperfusion, which was responsible for 8.5% of total variance (p = 0.015, Figure 4B). Genotype was responsible for 12.3% of variance of right lung MPO activity (p =0.015), and there was a significant interaction between genotype and ischemia-reperfusion (p = 0.019), accounting for an estimated 11.5% of total variance. Post-hoc comparisons identified a significantly lower MPO activity in the right lungs of both *Myd88* ^*-/-*^ mice and *Tlr4* ^*-/-*^ mice as compared to wildtype mice following ischemia-reperfusion.

### Lung permeability is reduced in Myd88^-/-^ mice as compared to C57BL/6 mice following ischemia-reperfusion

**Figure 5:**
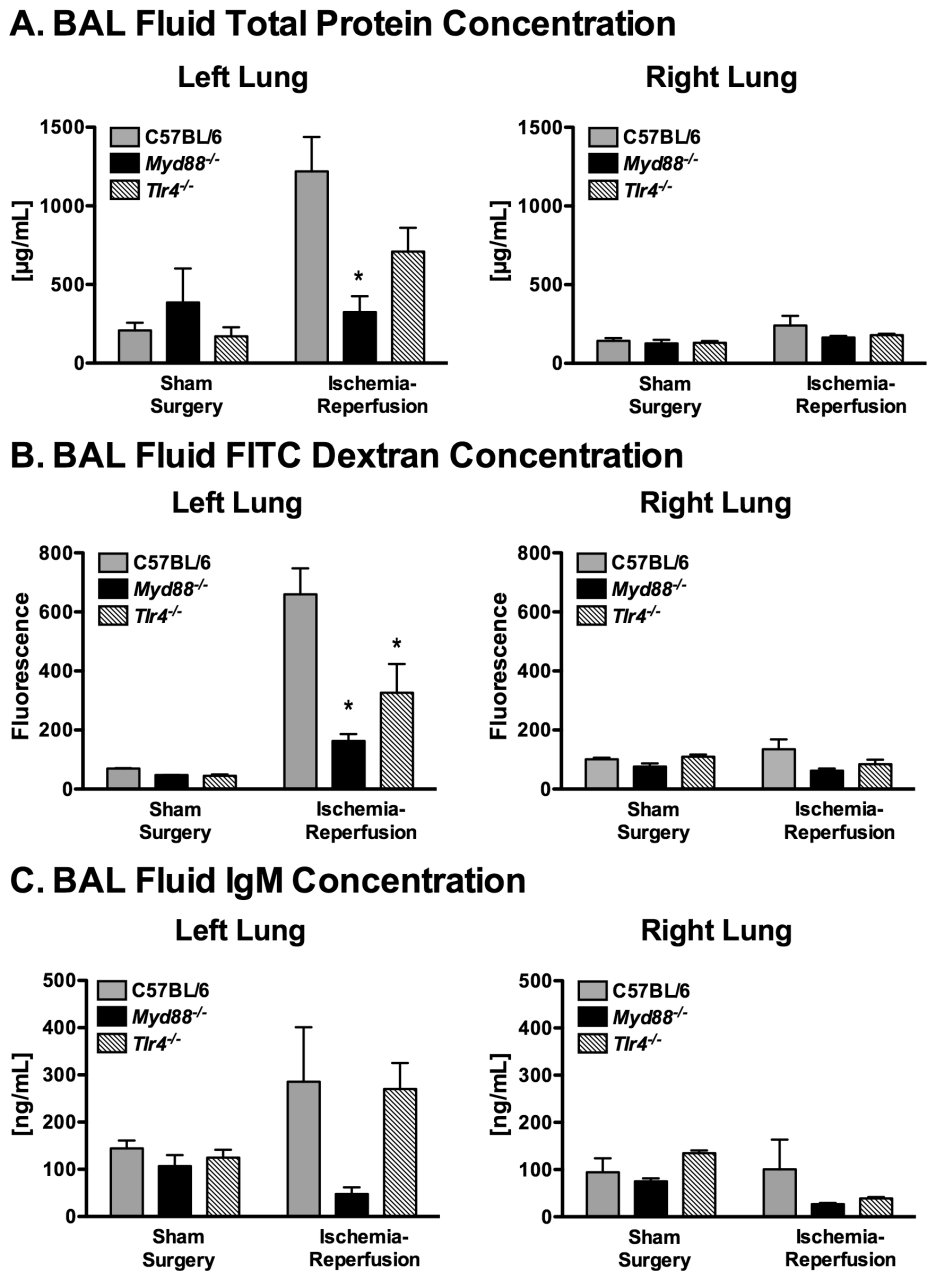
Lung permeability as measured by total protein (A), 70kD Dextran (B), and IgM (C) in bronchoalveolar lavage fluid. (n = 6/grp, *p ≤ 0.05 compared with C57BL/6 and, ^†^p ≤ 0.05 compared with *Tlr4* ^*-/-*^).

In a second series of experiments, mice received an intravenous injection of 100 µl of 10 µM FITC-dextran after one hour of reperfusion or after two hours of mechanical ventilation, following sham left thoracotomy. Three hours after FITC-dextran injection, mice were killed and the left and right lungs were individually lavaged to determine changes in alveolar-capillary barrier permeability. Six mice per genotype were analyzed following ischemia and reperfusion. Seven Myd88^-/-^ were subjected to the ischemia-reperfusion protocol, but BAL fluid could not be obtained on one mouse. Ischemia-reperfusion significantly affected permeability in the left lung as measured by BAL fluid total protein concentration (Figure 5A, p = 0.002, 28.2% contribution to overall variability) and by BAL fluid FITC-dextran (Figure 5B, p < 0.001, 33.3% of overall variability). A significant interaction effect between genotype and ischemia-reperfusion was also observed for both total protein concentration (p = 0.02, 21.1% of overall variability) and FITC-dextran amount (p = 0.02, 11.9% overall variability). Following ischemia-reperfusion, left lung BAL fluid concentrations of both total protein and FITC-dextran were significantly lower in *Myd88* ^*-/-*^ mice as compared with C57BL/6 mice; whereas, *Tlr4* ^*-/-*^ mice had intermediate protection from barrier dysfunction. Concentrations of the larger IgM multimer followed a similar pattern but the differences were not statistically significant (Figure 5C). In contrast to the left lung, permeability measures were no different in the right lung following left lung ischemia-reperfusion as compared to sham surgery controls and were similar among the different genotypes (Figure 5A-C).

### Ischemia-reperfusion releases a non-LPS TLR4 ligand into the alveolar compartment of the injured lung

These data suggest that lung ischemia followed by reperfusion results in production of pro-inflammatory endogenous ligands, which signal via a MyD88-dependent pathway. 

**Figure 6:**
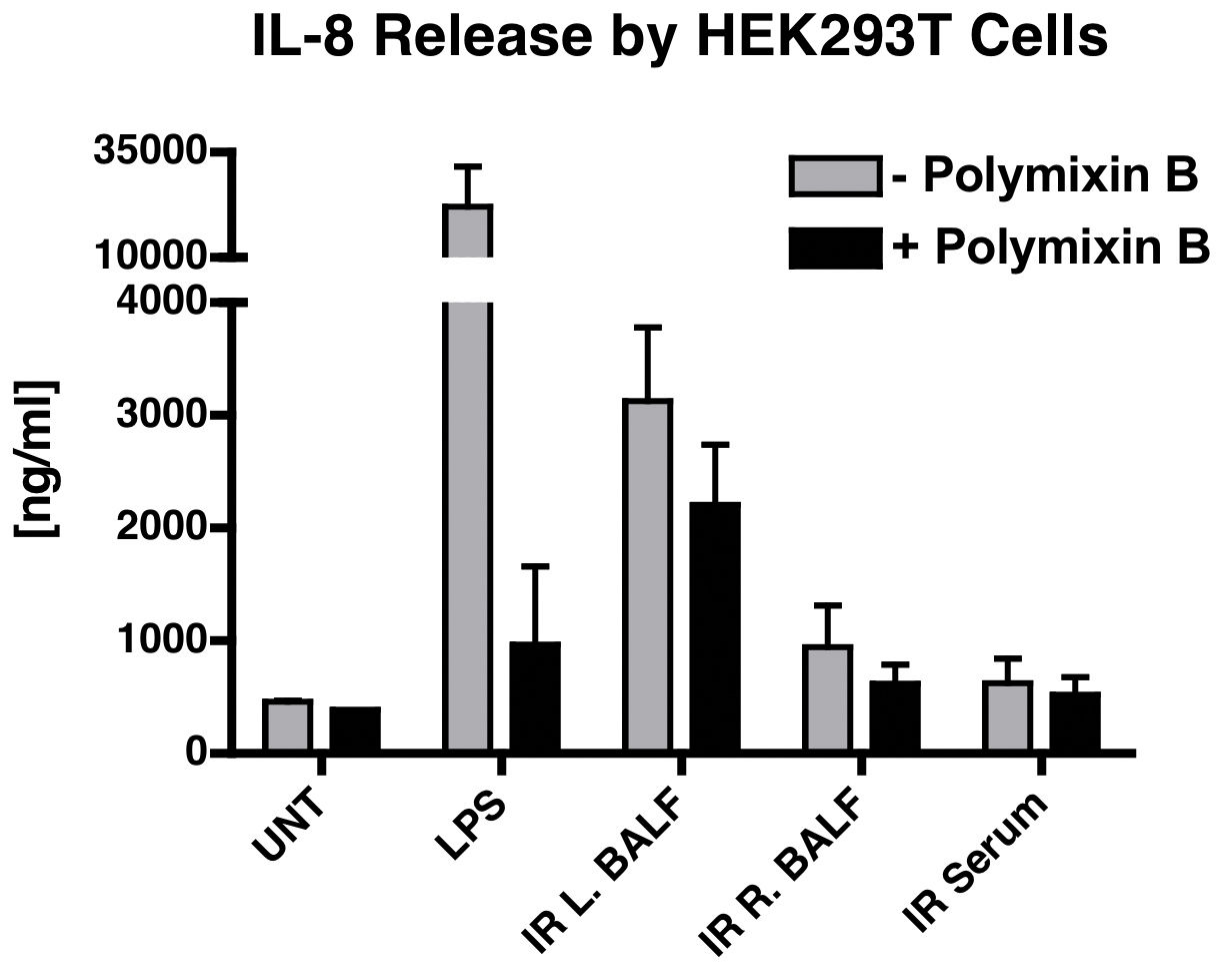
IL-8 release by HEK293T cells expressing murine TLR4, CD14, and MD2. Cells were stimulated with serum, right lung BAL fluid, or left lung BAL fluid collected from mice subjected to 1 hr of left lung ischemia followed by 4 hr of reperfusion. Cells were treated with medium only for a negative control or with LPS (10 ng/ml) for a positive control. Each experiment was carried out with and without polymixin B (50 µg/ml).

Because *Tlr4* ^*-/-*^ mice had partially attenuated MCP-1/CCL2 expression and permeability in response to ischemia-reperfusion, we speculated that tissue damage resulted in release of functional signals of tissue injury, i.e. DAMPs or alarmins [36]. TLR4 has been reported to be a primary receptor for these tissue damage signals [37,38]. To determine whether ischemia-reperfusion resulted in release of a molecule, which signals via TLR4, we stimulated HEK293T cells that stably express murine TLR4/CD14/MD2 with BAL fluid collected from the left lung following ischemia-reperfusion and measured resultant expression of IL-8. To exclude the possibility of a false positive signal from LPS contamination, experiments were performed in the presence and absence of polymixin B. To determine whether left lung ischemia-reperfusion resulted in systemic release of a TLR4 ligand, we also stimulated the HEK293T cells with right lung BAL fluid and with serum collected from the same mice. We found that stimulation of the HEK293T cells with left lung BAL fluid resulted in a significant increase in IL-8, which was only partially attenuated by the addition of polymixin B (3127±655 vs. 2205±536 ng/ml IL-8, p = 0.0125, Figure 6). In contrast, stimulation of the HEK293T cells with either right lung BAL fluid or serum resulted in significantly less IL-8 release (943±368 and 624±220 ng/ml, respectively) as compared with BAL fluid from the left lung (Figure 6). The difference between left lung BAL fluid and both right lung BAL fluid and serum was present regardless of the presence or absence of polymixin B. These data indicate that left lung ischemia and reperfusion resulted in release of a TLR4 ligand other than LPS. However, this ligand was not present at biologically active levels within the systemic circulation. The absence of a TLR4 activating component in the right lung BAL fluid indicates that this response requires lung ischemia and reperfusion and does not result from ventilator-induced injury, alone.

### Lung permeability following ischemia-reperfusion is attenuated when myeloid-derived cells lack MyD88

**Figure 7:**
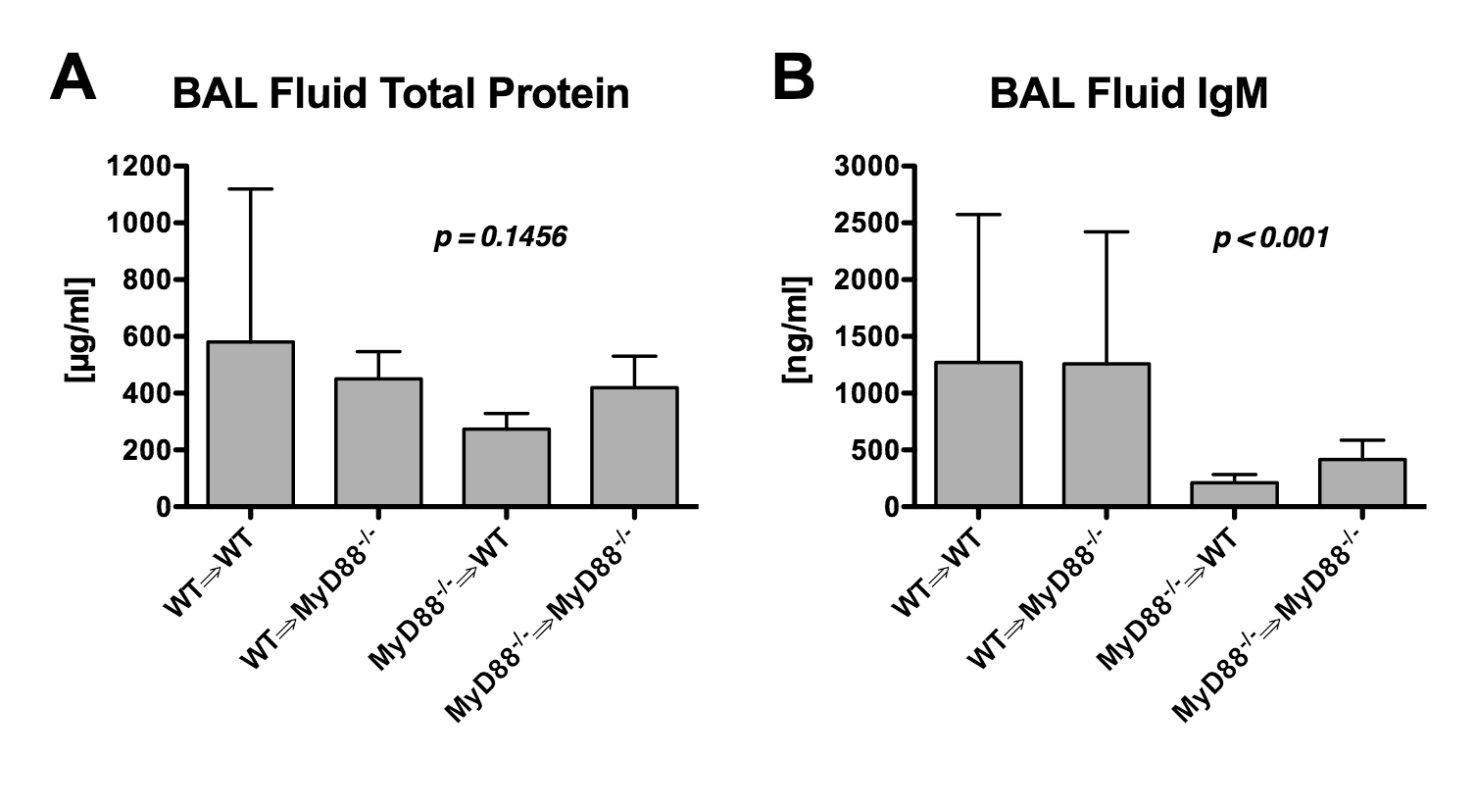
Chimeric mice expressing MyD88 in all cells (WT⇒WT), restricted to myeloid cells (WT⇒*Myd88* ^*-/-*^), restricted to non-myeloid cells (*Myd88* ^*-/-*^⇒WT), or in no cells (*Myd88* ^*-/-*^⇒*Myd88* ^*-/-*^).

To determine whether lung parenchymal cells or myeloid-derived cells such as alveolar macrophages and/or recruited leukocytes were responsible for MyD88-dependent permeability changes, we compared the responses of four different groups (n=5/grp) to left lung ischemia and reperfusion: 1) mice expressing MyD88 in all cells (wildtype marrow into wildtype mice WT⇒WT); 2) mice with MyD88 expression restricted to myeloid-derived cells (wildtype marrow into *Myd88* ^*-/-*^ mice or WT⇒*Myd88* ^*-/-*^); 3) mice with MyD88 expression restricted to non-myeloid cells (*Myd88* ^*-/-*^⇒WT); and 4) mice without MyD88 expression in any cells (*Myd88* ^*-/-*^⇒*Myd88* ^*-/-*^). Among the four groups, there was a trend towards lower protein concentration in the BAL fluid of mice lacking MyD88 in myeloid cells (*Myd88* ^*-/-*^⇒WT and *Myd88* ^*-/-*^⇒*Myd88* ^*-/-*^, Figure 7A). However, when evaluating permeability to very large IgM molecules, there was a significant difference among groups with much less IgM leakage into the alveolar space of mice lacking MyD88 expression in myeloid cells (Figure 7B). These data indicate that MyD88-dependent signaling in myeloid cells (e.g., alveolar macrophages and/or recruited leukocytes) contributes to the increased pulmonary leak observed with ischemia and reperfusion.

## Discussion

We hypothesized that lung inflammation and vascular barrier dysfunction following lung ischemia-reperfusion is mediated through MyD88-dependent recognition of endogenous ligands. The important findings of this study are that: 1) MCP-1/CCL2 expression and vascular barrier dysfunction are increased in the left lung following ischemia-reperfusion in mice via a MyD88-dependent mechanism; 2) *Tlr4* ^*-/-*^ mice display an intermediate phenotype, suggesting that multiple MyD88-dependent receptors are required for maximal injury following ischemia-reperfusion; 3) vascular barrier dysfunction with ischemia-reperfusion injury requires MyD88-dependent signaling on myeloid-derived cells but not on structural lung cells; and 4) left lung ischemia-reperfusion causes remote inflammatory changes in the right lung.

Systemic expression of MCP-1/CCL2 has recently been identified as an important risk factor for early primary graft dysfunction, the clinical manifestation of ischemia-reperfusion injury [7,8]. Our study implicates MyD88-dependent signaling in the early induction of MCP-1/CCL2 expression in the lung, following ischemia and reperfusion. Furthermore, *Myd88* ^*-/-*^ mice had attenuated vascular barrier disruption after ischemia-reperfusion injury. Finally, ischemia-reperfusion upregulation of KC/CXCL1 expression and neutrophil accumulation (as estimated by MPO activity) were lower in *MyD88* ^*-/-*^ mice, although these differences were not statistically significant in this study. These data strongly support a role for generation of endogenous ligands that induce inflammation and permeability changes via signaling by MyD88-dependent receptors such as the Toll-like receptors and the IL1 receptor family.

MyD88 is an adapter protein essential for signaling by all Toll-like receptors with the exception of TLR3 [16,39], suggesting that production of tissue damage-related endogenous TLR ligand(s) may be partially responsible for lung inflammation and injury following ischemia and reperfusion. Indeed, TLR4 has been implicated in ischemia-reperfusion injury of many organs including heart [40,41], lung [25], liver [42], and kidney [43]. Our data confirm a role for *Tlr4* ^*-/-*^ activation by one or more unidentified DAMPs in that BAL fluid from mice with ischemia-reperfusion caused TLR4-dependent production of IL8 in a HEK293 reporter cell line. Additionally, as compared to normal mice, *Tlr4* ^*-/-*^ mice had reduced neutrophil accumulation, MCP-1/CCL2 expression, and reduced vascular permeability as measured by FITC-Dextran leakage into the lung following ischemia-reperfusion injury. Interestingly, *Tlr4* ^*-/-*^ mice only partially recapitulated the protective effect seen in *MyD88* ^*-/-*^ mice. This incomplete protection observed in *Tlr4* ^*-/-*^ mice indicates that alternative MyD88-dependent pathways contribute to lung injury in our model. TLR2 has been implicated as a receptor for endogenous ligands associated with tissue damage. In bleomycin-induced and hyperoxia-induced models of lung injury, hyaluronan degradation products have been associated with inflammation, and disruption of both TLR2 and TLR4 are required to fully recapitulate the protection seen with absence of MyD88 [44]. Endogenous ligands for MyD88-dependent TLR-7, -8, and -9 have also been reported [45] although their relevance to lung injury is unknown. Other MyD88-dependent receptors such as the IL1 family of receptors may also play an important role in the full development of ischemia-reperfusion injury. Future studies targeting different combinations of receptors will be required to fully characterize the relevant MyD88-dependent receptors contributing to ischemia-reperfusion injury.

Unique among known TLRs, TLR4 signals via two parallel pathways, one that requires the MyD88 adapter protein and the other that requires the TIR-domain-containing adapter-inducing interferon-β (TRIF) adapter protein. TRIF-dependent, MyD88-independent TLR4 signaling has been implicated in several models of lung injury other than ischemia-reperfusion [46]. One prior study has evaluated the role of MyD88-dependent TLR4 signaling in lung ischemia-reperfusion injury [26]. Zanotti and colleagues found that HeJ mice, which lack TLR4, had attenuated lung permeability after ischemia-reperfusion and subsequently compared *Myd88* ^*-/-*^ mice with C57BL/6 mice (the background strain for the *Myd88* ^*-/-*^ mice). They found that *Myd88* ^*-/-*^ mice had similar permeability as the C57BL/6 wildtype mice, suggesting that protection in the TLR4-deficient HeJ mice was mediated by the MyD88-independent, TRIF-dependent pathway. The authors did not report inflammation or leukocyte recruitment in this study. The reasons for the discrepancy between our current results and this prior study are unknown; however, there were several technical differences between the two studies, which could plausibly contribute to the differences. In the prior study, larger tidal volumes were used, and these were not reduced during single right lung ventilation, potentially resulting in an augmented contribution of ventilator-induced lung injury. Additionally, reperfusion times were generally shorter in the prior study; therefore, it is possible that MyD88-dependent signaling becomes more relevant as reperfusion times increase. Although we did not directly evaluate the role of TRIF-dependent TLR4 signaling in our model, overall signaling via MyD88-dependent pathways clearly contributed more to injury than TRIF-dependent pathways given the greater protection in *Myd88* ^*-/-*^ mice as compared to *Tlr4* ^*-/-*^ mice.

An interesting additional finding from our study is that there is significantly increased MCP1/CCL2 expression and MPO activity in the right lung following left lung ischemia-reperfusion and that this right lung response is attenuated in *Myd88* ^*-/-*^ and, to a lesser extent *Tlr4* ^*-/-*^ mice. One theoretical explanation for this finding is that the right lung could have been subjected to volutrauma and ventilator-induced lung injury during left hilar clamping. To reduce this possibility, we decreased tidal volumes to 6 mL/kg during the left lung ischemic time and increased respiratory rate to compensate. The fact that peak airway pressures were, if anything, higher in the sham surgery groups as compared to the ischemia-reperfusion groups during the ischemic period (Figure 1), suggests that the increased inflammation seen in the right lung was due to remote sequelae of left lung ischemia-reperfusion. This led to the speculation that MyD88-dependent ligands may be released into the systemic circulation resulting in distal organ inflammatory responses. However, we did not find evidence of a TLR4 ligand in the serum or in the BAL fluid of the right lung, suggesting that an endogenous TLR4 ligand was not disseminated into the systemic circulation. Alternatively, the amount of an endogenous TLR4 ligand may have been below the detection limit of the reporter cell assay and/or the timing of sample collection missed the peak levels of circulating ligand. The clinical significance of these findings is unclear. It is possible that, with a more prolonged reperfusion period, the right lung may have developed progressive lung injury. Certainly, primary graft dysfunction can occasionally be associated with ARDS in a native, contralateral lung. Our data suggest that one or more circulating DAMPs may be responsible for this phenomenon; however, further studies to confirm this finding and identify the relevant receptors are required. 

An important caveat of this study is the use of in situ warm ischemia as opposed to *ex vivo* cold ischemia present with actual lung transplantation. It is possible that actual organ transplantation may result in a different pattern of inflammatory activation. Because of the requirement for genetically modified mice in this study and the technical challenges of microsurgery required for actual lung transplantation in mice, we have used the common model of in situ warm ischemia.

### Conclusions

Lung ischemia-reperfusion causes inflammation and permeability changes in a MyD88-dependent manner. Although endogenous ligands to TLR4 contribute to lung ischemia-reperfusion injury, the protective effect in *Tlr4* ^*-/-*^ mice on MCP-1/CCL2 expression and vascular barrier dysfunction were relatively small compared to that seen in *Myd88* ^*-/-*^ mice. These data suggest that identifying additional MyD88-dependent pro-inflammatory pathways relevant to ischemia-reperfusion could promote development of new therapeutic modalities to attenuate lung injury following lung transplantation. Additionally, combination therapy with anti-TLR4 agents and treatment directed at other MyD88-dependent receptors may provide optimal protection against ischemia-reperfusion injury.
